# Editorial: Green chemistry to recycle Fe/C-rich wastes for environmental pollution control

**DOI:** 10.3389/fchem.2023.1178615

**Published:** 2023-04-13

**Authors:** Zhu Suiyi, Fan Wei, Xie Xinfeng, Jadambaa Temuujin

**Affiliations:** ^1^ College of Resources and Environment, Zhongkai University of Agriculture and Engineering, Guangzhou, China; ^2^ School of Environment, Northeast Normal University, Changchun, China; ^3^ College of Forest Resources and Environmental Science, Michigan Technological University, Houghton, MI, United States; ^4^ School of Advanced Study, CITI University, Ulaanbaatar, Mongolia

**Keywords:** green chemistry, Fe/C-rich wastes, wastewater treatment, resource reusage, regeneratable materials

Iron (Fe) and carbon (C) are two of the most common elements which exist in many industrial wastes and byproducts causing various environmental problems. Traditionally the Fe/C-rich wastes were recycled in most cases for Fe- and C-containing chemicals using chemical-biological processes or used as additives in smelting, building materials, and biomass fuels. However, there are a large number of Fe/C-rich wastes have been landfilled. Much research is needed to effectively and efficiently use the Fe/C-rich wastes and byproducts as a resource for products either as a unmodified or modified material.

Recent advances in modern chemistry have provided new solutions for utilization of Fe/C-rich wastes, especially in the field of environmental remediation and pollutants control. With advanced chemical treatments, the Fe/C-rich wastes can be converted to value-added materials and products, such as adsorbents, flocculants, catalysts, precipitants, demulsifying reagents, conditioning reagents, filter materials, and membranes. These materials and products have been extensively used in treating waste streams and mitigating the environmental impacts of spills and pollutants.

This Research Topic collected the state-of-art green chemistry in recycling Fe/C-rich wastes for materials and products that can be used in environmental remediation and pollutants. A total of five papers were included in this issue to cover a broad spectrum of advanced Fe/C-bearing materials, ranging from Fe-encapsulated biochar, carbon nanotube membranes, and Fe-oxides modified air stone, to K_2_FeO_4_ chemicals. These papers introduced the emerging method for surface modification, the new routes for Fe/C conversion, and the new insights into the interfacial electron transfer. The materials produced from the Fe/C-rich wastes using advanced green chemistry showed great potential in removal of organics and heavy metals through activated ozone and peroxodisulfate and in direct adsorption of antibiotics and antibiotic resistance genes from wastewater streams.

With the application of advanced green chemistry, there are three topic areas in the recycling of Fe/C-rich wastes deserve further studies: 1) The *in-situ* phase transition of Fe-bearing minerals in Fe/C-rich wastes and the induced interface catalytic reactions for enhanced removal of contaminants; 2) The high-value products regenerated from the Fe/C-rich wastes, and the related technologies, mechanisms, and applications; 3) the closed-loop utilization of energy and resources in the recycling of Fe/C-rich wastes to achieve near-zero release.

The advancement in green chemistry has bring made significant progress in utilization of Fe/C-rich wastes and it will continue to benefit our society by reducing waste discharges and the consumption of non-renewable resources. The recycling of Fe/C-rich wastes for environmental remediation and pollutants is an important part of “using wastes to treat wastes” and will make a significant contribution to the circular economy.

